# Neurophysiological correlates of linearization in language production

**DOI:** 10.1186/1471-2202-9-77

**Published:** 2008-08-05

**Authors:** Boukje Habets, Bernadette M Jansma, Thomas F Münte

**Affiliations:** 1 School of Psychology, University of Birmingham, Birmingham, UK; 2 Department of Cognitive Neuroscience, Maastricht University, Maastricht, The Netherlands; 3 Department of Neuropsychology, Otto-Von-Guericke Universität Magdeburg, Magdeburg, Germany

## Abstract

**Background:**

During speech production the planning of a description of several events requires, among other things, a verbal sequencing of these events. During this process, referred to as linearization during conceptualization, the speaker can choose between different types of temporal connectives such as 'Before' X did A, Y did B' or 'After' Y did B, X did A'. To capture the neural events of such linearization processes, event-related potentials (ERP) were measured in native speakers of German. Utterances were elicited by presenting a sequence of two pictures on a video screen. Each picture consists of an object that is associated with a particular action (e.g. book = reading). A coloured vocalization cue indicated to describe the sequence of two actions associated with the objects in chronological (e.g. red cue: 'After' I drove the car, I read a book) or reversed order (yellow cue).

**Results:**

Brain potentials showed reliable differences between the two conditions from 180 ms after the onset of the vocalization prompt, with ERPs from the 'After' condition being more negative. This 'Before/After' difference showed a fronto-central distribution between 180 and 230 ms. From 300 ms onwards, a parietal distribution was observed. The latter effect is interpreted as an instance of the P300 response, which is known to be modulated by task difficulty.

**Conclusion:**

ERPs preceding overt sentence production are sensitive to conceptual linearization. The observed early, more fronto-centrally distributed variation could be interpreted as involvement of working memory needed to order the events according to the instruction. The later parietal distributed variation relates to the complexity in linearization, with the non-chronological order being more demanding during the updating of the concepts in working memory.

## Background

Psycholinguistic models of speech production distinguish between four major processing stages to transfer an idea into a meaningful utterance [1-5], involving conceptual, syntactic, phonological encoding and articulation.

Evidence for these different levels has been obtained from speech errors and picture naming studies (for a review see [6]). In addition to the question as to what stages can be distinguished during language production, it has also been of interest when these different types of information become available. Electrophysiological measures have been particularly helpful in addressing this question [7-12].

Existing psycholinguistic theories are mainly based on single word production. Hence, many aspects are discussed related to conceptual representation and selection of single words and as a consequence, little empirical evidence is available for the conceptual planning in complex utterances [13-16]. The present study aims to investigate the neural aspects of conceptualization during speech planning, i.e. the streamlining of ideas into meaningful utterances.

From a psycholinguistic perspective, conceptualization involves two steps, usually referred to as macro – and micro-planning [17]. During macro-planning, an intention or goal is chosen, divided into sub-goals that are planned, ordered and specified for intended mood and content. Its output is an ordered sequence of speech-act intentions (often shortened to speech acts, SA). These speech acts are further specified during the micro-planning phase, where they are assigned particular informational structures (e.g. what should be expressed as topical, focussed, or new information) and perspective. Note that these two processes do not necessarily have to be as separated and serial as is described here. It is very well possible that micro-planning already starts before macro-planning processes are completed.

The goal of the present study was to gain more insight in macro-planning processing and, to be more specific, in the processes underlying the ordering of events ('the linearization problem').

The speaker's decision to verbally order events in a particular way is influenced by the speech context in many ways. One such ordering principle is chronological order. Usually, speakers prefer a chronological order of event sequence, for example, 'I woke up this morning and ate breakfast'. But there are situations in which a speaker might choose against a chronological order, for example when the event is the most salient one and is therefore mentioned first ('I got fired when I arrived at work'), a process referred to as 'topicalization' [17].

The speaker can order the events by using temporal connectives available in the language, such as 'before' and 'after'. These are linguistics signals informing the comprehender about the order of the upcoming events. 'After' (on a sentence-initial position) indicates that events will be described in the actual order of occurrence, whereas 'before' signals a reversal (e.g., 'After I ate dinner, I did the dishes' or 'Before I did the dishes, I ate dinner').

Evidence that a chronological order is preferred to non-chronological order comes from language acquisition and language disorder studies. When asked to act out 'Before/After' instructions, children usually have more difficulty acquiring 'Before' than 'After' [18-20]. For example, Stevenson and Pollitt [21] tested the understanding of temporal terms of English children aged 2 to 5 by letting them act out situations described by sentences containing the words 'Before' and 'After'. Children showed a tendency to act out only the first clause of 'Before' sentences. This suggests that they do not understand the reversed temporal relation between events that is depicted by 'Before' sentences and the authors concluded that the children had greater difficulty understanding 'Before' constructions in comparison to 'After' sentences [22].

Parkinson patients have also been shown to make more errors for sentences starting with 'Before', since they tend to understand 'Before' sentences as if they had started with 'After' [23]. In a related study [24], Parkinson patients also failed to understand so-called object-relative clauses (in which the subject of the main clause serves as the object of the relative clause). These sentences were interpreted as subject-relative clauses, i.e. the subject of the main clause was also assumed to be the subject of the relative clause. Because of fewer filler/gap positions in subject relative clauses, these sentences are less demanding on working memory. Taken together, it appears that the understanding of non-chronological order sentences is more difficult for Parkinson patients because these sentence constructions require more working memory processing.

The role of working memory in the processing of temporal connectives has been investigated by event-related brain potentials (ERPs) [25]: Participants read sentences that started with the temporal connectives 'Before' and 'After'. The sentences appeared one word at a time whilst EEG was recorded (e.g.; 'Before/After the psychologist submitted the article, the journal changed its policy'). 'Before' sentences differed from 'After' sentences by a ramp-like negativity which started around 300 ms after onset of the sentence's initial word and lasted for the entire sentence. This 'Before/After'-difference was greater for those participants with better individual working memory capacity, indicating an immediate interaction (already at 300 ms after presenting 'Before/After' words) between working memory and linearization of conceptual events. The authors concluded that sentences containing a non-chronological order of events are more demanding on working memory than chronological order sentences, possibly leading to different discourse representations for the two types of sentences.

In sum, these studies show that non-chronological order constructions (sentences starting with 'Before') seem to be more difficult to understand than chronological order constructions (beginning with 'After'). Furthermore, it seems that the difficulty with 'Before' sentences is due to higher verbal working memory load. The aim of the present paper is to investigate whether this difference in linearization complexity is also reflected in language production. Is it plausible to expect a similar complexity effect in production, or would the mere fact of producing, hence choosing a temporal relation between events extinguish possible differences in difficulty?

Speech production is achieved with amazing speed, going from the initial planning stage to articulation in just a few hundred milliseconds. If one intends to capture the neural events involved in speaking as they unfold in time, electrophysiological measures are the method of choice. A number of ERP studies have used surrogate tasks (i.e. mapping of specific semantic, syntactic, or phonological features of the word corresponding to a picture to one or two button-press decisions) to study information availability of linguistic information [7,10,11,26-29]. Some have used delayed vocalization in order to avoid possible speech artefacts. Most recently, overt vocalization tasks in ERP studies [30,31] have shown that reliable and artifact-free ERPs can be generated in the interval between a stimulus onset and the respective vocalization of an overt utterance.

The present study uses this method to investigate the conceptual planning of chronological and non-chronological order constructions. Moreover, the ERP analysis in the present study focus on a relatively smaller time window (until 600 ms after stimulus onset) to minimize possible interference of speech and other artefacts, but also to reduce the influence of speech preparation. Subjects saw a sequence of two pictures, followed by a cue. They were instructed by the colour of that cue to utter, in German, a sentence describing the typical actions starting with the temporal connectives 'Before' or 'After'. An example for a chronological order would be 'Nachdem ich fahre, lese ich', ['After I drive (a car), I read (a book)']. A non-chronological order would be: 'Bevor ich lese, fahre ich', ['Before I read (a book), I drive (a car)']). The proportion of each utterance format was 50% and it was randomized across blocks.

As we supposed that the two conditions differ in working memory load, we expected to find ERP differences related to this fact (paralleling the results of Münte et al. [25] in the comprehension domain).

## Results

### Behavior

Voice onset latencies (VOL) were collected by means of a voice key (Presentation, version 9.10). The responses of seventeen subjects were averaged and included in the analysis. The overall amount of errors for each subject was less than 10 percent. There was no significant difference in the error proportions of the two conditions (mean chronological order = 4.9, mean non-chronological order = 5.8, (t(16) = -.74, p = .47) Also, no significant difference in onset latency were observed (mean VOL = 1362 ms for both conditions).

### ERPs

**Figure 1:**
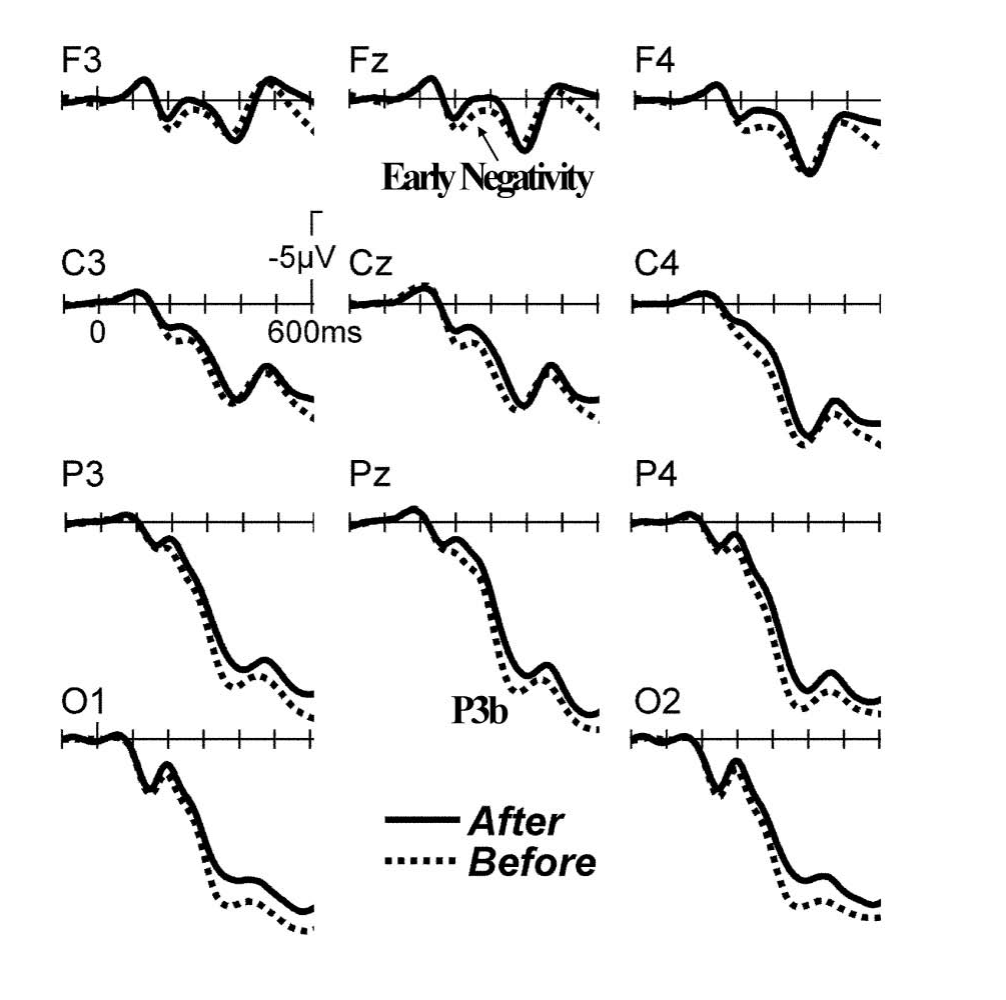
**Grand average ERPs at selected scalp sites time locked to the onset of the coloured fixation cross which prompted the utterance.** The Before condition gave rise to a more positive waveform starting at about 180 ms.

The grand average ERPs time-locked to the fixation cross are shown in Figure 1.

**Figure 2:**
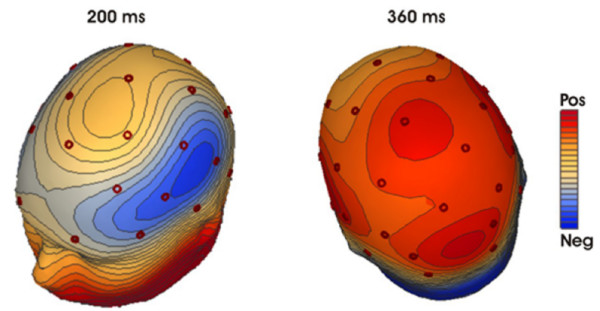
**Spline interpolated isovoltage maps of the difference between the After and Before condition.** During the first phase a fronto-central distribution is evident, while beyond 350 ms a clear parieto-occipital maximum emerges (min/max scaling: -0.24 to 0.24 μV at 200 ms; -0.98 to 0.98 μV at 360 ms).

The two conditions started to diverge around 180 ms, with the 'Before' condition being more positive than the 'After' condition in the entire time window. This difference appeared to have two different portions as is illustrated by the isovoltage maps in Figure 2: The earlier portion around 200 ms had a fronto-central distribution, whereas the later portion around 350 ms had a more posterior distribution.

For the early time-window (180–230 ms), reliable differences between 'Before' and 'After' sentences were revealed by main effects of the factor Order for midline (F(1,16) = 4.83, p < 0.05), parasagittal (F(1,16) = 6.22, p < 0.025) and temporal (F(1,16) = 9.09, p < 0.01) electrode sites. No reliable interactions were obtained between Order and the topographical factors (see Table 1).

**Table 1:** F-values from ANOVAs comparing the different conditions at temporal, parasagittal and midline electrode locations.

	***Df***	**F**	**180–230 ms**	**350–400 ms**
*Temporal*				
Type of Order (O)	1,16	9.09	.01	
O × Ant	2,32	9.96		.005

*Parasagittal*				
Type of Order (O)	1,16	6.22	.025	
O × Ant	4.64	7.12		.003

*Midline*				
Type of Order (O)	1,16	4.83	.05	

For the later time-window (350–400 ms), a significant Order by Anterior/Posterior interaction for the parasagittal (F(4,64) = 7.12, p < 0.003) and temporal sites (F(2,32) = 9.96, p < 0.005) reflected the fact that during this time window a posteriorly distributed difference between 'Before' and 'After' sentences was present (see Figure 2). Order did not reach significance (F(1,16) = 1.24/2.02/1.88; all p > .05) for midline/parasagittal/temporal electrode sites, see Table 1).

To test whether indeed the early and late portions of the 'Before/After' differences had different distributions, we determined the mean amplitude of the 'After' minus 'Before' difference waves in the 180–230 ms and 350–400 ms time-windows for all 29 scalp electrodes. These values were subjected to the vector normalization procedure described by McCarthy and Wood [32] and then entered into an ANOVA with time-window (early vs. late) and electrode site (29 levels) as factors. A significant interaction between time-window and site (F(28,448) = 3.06, original p < 0.0001, Huynh-Feldt corrected: p = 0.038) indicated that the difference between condition had indeed a different distribution in the two time-windows.

## Discussion

The present study investigated conceptualization processes while subjects generated a verbal description of two events in a chronological and non-chronological order sequence. The ERP results revealed differences between 'Before/After' sentences in terms of an early fronto-central negativity for 'After' versus 'Before' sentences. This effect is followed by a later parietal positivity for 'Before' in comparison to 'After' sentences. Further, no difference in voice-onset latency (VOL) was found between the two order constructions.

The observed lack of effects for VOL might seem counterintuitive at a first glance, as one would assume longer latencies for the presumably more difficult planning in the 'Before' case. Note that VOL measures the end of an entire information processing sequence. Strategic effects and execution are included next to the process of interest, i.e. the conceptual planning. However, this result may also be related to a ceiling effect or a strategy to delay naming until the entire utterance plan is available.

Whereas the VOL are not informative in this case, ERP results show that the brain clearly distinguishes between sentences in which a sequence of events is uttered in chronological order or non-chronological order. An early fronto-central difference (180–230 ms after cue onset) differentiated between 'Before' and 'After' sentences. This result is in line with previous fMRI studies showing that the prefrontal cortex plays a critical role in temporal sequencing [33-35]. Moreover, patients with prefrontal lesions are known to be impaired in generating and evaluating order of series and actions [36,37]. More specifically, a fronto-temporal network involved in verbal semantic memory decision or categorization processes has been found in several studies before [38,39].

In parallel to an earlier comprehension study [25] using sentences with temporal connectives which had shown an interaction between working memory and linearization processes reflected in a frontal negativity, it seems plausible to interpret the early negativity in the context of working memory. Indeed, several studies looking at the comprehension of various syntactic sentence structures (SO and SS relative clauses) revealed an anterior negativity for the more complex sentence structures (SO sentences) [40,41], which was interpreted in terms of more working memory demands for the understanding of more complex sentence structures.

The present study reveals a higher negativity for chronological order sentences. This might seem counterintuitive, as it implies that working memory demands are higher during the conceptualization of a chronological order sentence. The direction of this effect can be explained by taking a closer look at the setup of the experiment. The object pictures were presented sequentially during this study, meaning that the first picture needed to be kept in mind longer when subjects had to create a chronological order sentence. This is contrary to the production of the non-chronological order that started with the utterance of the last presented object. Taken together, in order to utter a chronological order sentence, subjects had to go back two steps to start with the first presented object and this process naturally appeared to demand more working memory processing.

Further, we found a parietal positivity between 350 and 400 ms for the before condition. Its distribution and polarity suggest that this may be an instance of the P300, or P3b. The P300 is a well-known component often found in tasks investigating attention devoted to a stimulus, stimulus salience, task relevance, objective and subjective probability among a stimulus sequence, or the amount of resources needed to process a stimulus [12,42-45].

In this light, the greater P3b for 'Before' constructions in the present study may reflect greater attentional processing in terms of 'context maintenance' processes. A recent study from our laboratory tapping into process-related strategies during conceptualization, found a similar parietal positivity. As described in the introduction, process-related strategies are used when content-related information (e.g. differences in time between events, as used during content-related strategies) is not available. In this experiment, process-related strategies were investigated by manipulating the complexity of utterances describing the direction of an arrow in a network of geometrical forms (easy: downwards, medium: downwards to the triangle, complex: downwards to the grey triangle) [46]. In this case, medium and complex utterances were associated with a parietal positivity when compared with the "easy" condition.

To sum up, the present study showed that conceptualization of 'Before' and 'After' order sentences leads to more conceptualization processing when non-chronological order constructions are being built. This finding is in line with language comprehension studies that have shown that these types of sentences are more difficult to understand, and with a second study in our laboratory that investigated complexity differences in process related strategies. Moreover, both the frontal and the parietal effect are in line with a fronto-temporal network found in fMRI studies looking at verbal semantic categorization processes [38,39].

The result is explained in terms of the P3b component reflecting attentional processing. In addition, this component might not reflect attention related processes per se, but rather a more general demand of resources required for stimulus processing. Seeing that 'before' sentences are the more complex sentences in our study, one could argue by extension that conceptualization in this case takes up more resources. A remaining question, at this point, is what the exact nature of the conceptualization difference is that we found in the present study. Whereas, in daily life, topicalization occurs when one event bears more significance in relation to another event, the current study's use of neutral items made events less salient and therefore, might have affected the conceptualization process of non-chronological orders. While we cannot exclude this line of reasoning entirely at this stage, the lack of difference in voice onset latency and error results for both conditions seem to speak against that possibility.

Although it is reasonable to suspect that this finding reflects the inverted narration relation between events that are expressed in non-chronological order constructions, further experiments are needed to address this question in more detail.

## Conclusion

In this ERP study addressing conceptualization processes during language production, a frontal negativity likely associated to greater working memory demands for chronological order constructions was found, which can be explained by the fact that the first event needs to be retrieved from working memory. Importantly, as in Marek et al [46], a parietal positivity was found for the more difficult (Before) condition, which appears to reflect effects of conceptualization complexity.

## Methods

All procedures were approved prior to the study by the ethics committee of the University of Magdeburg, which ensured compliance with the Helsinki Declaration.

### Participants

Thirty-two right-handed, neurologically healthy students aged between 20 and 32 (mean age 23.3, 23 women, 9 men) with normal or corrected to normal vision and German as their native language gave informed consent and were paid for their participation. Subjects with more than 25% loss of trials caused by blinking or movement artefacts were excluded from further analysis. In this study, artefacts were mainly caused by subjects not being able to sit still during talking, or most importantly, by movements prior to vocalization, i.e. in the time-window of interest related to speech planning prior to articulation. It was not uncommon for subjects to move their head or to blink immediately before they started producing an utterance. Some participants could not control these movements and to ensure clean recordings during the conceptualisation window, we excluded 15 subjects, leaving 17 subjects for the final analyses.

### Stimuli and procedure

A total of 75 pairs of black and white line-drawings (picture data base of the Max-Planck Institute for Psycholinguistics, Nijmegen; [47]) were used. Pictures were edited with Corel Draw version 11.0 to have the same resolution (300 × 300 dpi), size (33 × 33 mm) and colour combination (black on white background). Pictures were combined into pairs such that no semantic and phonological overlap between the words denoting the objects on the two pictures occurred.

Each picture pair was presented twice in each condition ('Before'/'After') with the position of the pictures switched. This resulted in a total of 150 picture pairs per one condition and 300 picture pairs for the entire experiment.

Each trial comprised the following sequence: The first object picture was presented for 500 ms, followed by a blank screen for 200 ms. This was replaced by a second object picture, presented also for 500 ms, followed by a coloured fixation cross with a duration of 5000 ms to allow for the overt response. At the end of each trial, a blank screen was shown for 500 ms which prepared the subjects for the next trial. Instructions were to assume the action 1 required for object 1 as having occurred first, while the action 2 associated with object 2 happened subsequent to action 1. Further, subjects were told to start their utterances with 'Before' or 'After'. The color of the fixation cross (red or yellow) specified whether participants generated the event description by means of a (chronological) 'After' or an (non-chronological) 'Before' sentence. For example, subjects saw the object 'book' and then the object 'couch', followed by a red fixation cross. The instructed, correct German utterance would then be 'Nachdem ich lese, sitze ich' [in English; 'After' I read (a book), I sit (down on the couch)']. The same objects followed by a yellow fixation cross, would require the utterance 'Bevor ich sitze, lese ich' [in English; 'Before' I sit (down on the couch), I read (a book)'; information in parentheses for clarification only]. In order to optimally match the two types of utterances, subjects were instructed to use an identical structure for both sentences (except for the initial word). They had to always produce both sentence parts in the present tense. The aim was to minimize variability among the answers, to keep overt production time as short as possible, and to avoid possible differences between the conditions due to lexical effects. Utterances were recorded to check for all of the above mentioned points. The presentation of the fixation cross color was randomized and counterbalanced over the two utterances. Subjects were instructed to utter the required sentence as soon and as correct as possible after the appearance of the cue. After the application of the EEG electrodes, subjects were seated in a sound-proof cubicle and received detailed explanations about their task. They received three practice runs before the actual experiment started. During the first practice run, subjects saw the pictures of the objects with the target verb written below the picture. They were asked to learn the verb-object association (For example, the object picture was 'book', accompanied by the verb 'reading'). The second practice run then showed the same pictures without presentation of the verbs and subjects had to name the pictures out loud. For these two practice sessions, subjects could take as much time as required and they had to perform errorless before continuing with the last practice session. The last session entailed 20 example trials of the experiment itself, so that subjects could familiarize themselves with the timing of the stimuli. Subjects were told to sit as still as possible, and to blink only while they were speaking.

### Recording and analysis

EEG was recorded with tin electrodes mounted in an electrode cap FP1, FP2, F3, F4, C3, C4, P3, P4, O1, O2, F7, F8, T7, T8, P7, P8, Fpz, Fz, Cz, Pz, Fc1, Fc2, Cp1, Cp2, Po3, Po4, Fc5, Fc6, Cp5, Cp6 positions of the 10/20 system. Two additional electrodes were placed at the left and right mastoid for referencing. The electrode placed on the left mastoid was used for online referencing. Data were re-referenced off-line to the mean of the activity at the two mastoid electrode sites. Vertical eye movements were measured with a bipolar montage comprising electrodes placed above the left eyebrow and below the left orbital ridge. Horizontal eye movements were measured with two electrodes placed at the left and right external canthi. EEG-data were recorded continuously (time-constant 10 seconds, filter settings 0.05 to 30 Hz) with a sampling rate of 250 Hz. Electrode impedances were kept below 5 kΩ. EEG was averaged time-locked to the onset of the colour cue for epochs of 700 ms including a 100 ms pre-stimulus baseline. For eye-blink rejection the maximum difference was set on 150 μV (8 subjects) or 200 μV (9 subjects) for the vertical EOG channel. Shifts were corrected with linear regression. The threshold for shift correction was set to 300 μV/s. Rejections were 25% on average, and there was no difference between the conditions. Only single trials free of blink and movement artefacts were included in the averages. To quantify the ERP effects, mean amplitudes were measured at midline (Fz, Cz, Pz), parasagittal (Fp1/2, F3/4, C3/4, P3/4, O1/2), and temporal (F7/8, T7/8, P7/8) sites in an early (180–230 ms) and a later (350–400 ms) time-interval. These were subjected to repeated measures ANOVA. Factors were Order (After versus Before), Anterior/Posterior (3 levels for midline and temporal, 5 levels for parasagittal) and Hemisphere (left versus right, the factor was not used for midline analyses). The Huynh-Feldt correction for inhomogeneities of covariance was used when appropriate. We report the corrected p-value in conjunction with the original degrees of freedom.

## Abbreviations

ERP: event-related potential.

## Authors' contributions

BH co-designed the study, analysed and performed the experiments and the statistical analyses and co-wrote the manuscript. BMJ and TFM co-designed the study and co-wrote the manuscript. All authors read and approved the final manuscript.
